# Correlation of PD-L1 Expression of Tumor Cells with Survival Outcomes after Radical Intensity-Modulated Radiation Therapy for Non-Metastatic Nasopharyngeal Carcinoma

**DOI:** 10.1371/journal.pone.0157969

**Published:** 2016-06-24

**Authors:** Victor H. F. Lee, Anthony W. I. Lo, Chun-Yin Leung, Wai-Hung Shek, Dora L. W. Kwong, Ka-On Lam, Chi-Chung Tong, Chun-Kin Sze, To-Wai Leung

**Affiliations:** 1 Department of Clinical Oncology, Li Ka Shing Faculty of Medicine, The University of Hong Kong, Hong Kong, Hong Kong; 2 Anatomical Pathology, Queen Mary Hospital, Hong Kong, Hong Kong; 3 Li Ka Shing Faculty of Medicine, The University of Hong Kong, Hong Kong, Hong Kong; Taipei Medical University, TAIWAN

## Abstract

**Purpose:**

We investigated if programmed death-ligand 1 (PD-L1) expression levels were prognostic of survival outcomes after intensity-modulated radiation therapy (IMRT) for non-metastatic nasopharyngeal carcinoma (NPC).

**Methods and Materials:**

104 patients with non-metastatic NPC treated with radical IMRT were investigated for their PD-L1 expression by immunohistochemistry (IHC) which were correlated with survival endpoints including locoregional failure-free survival (LRFFS), progression-free survival (PFS), distant metastasis-free survival (DMFS) and overall survival (OS).

**Results:**

After a median follow-up of 7.6 years, 21 (20.2%), 19 (18.3%) and 31 (29.8%) patients suffered from locoregional failure, distant metastases and overall disease progression, respectively, and 31 (29.8%) patients died. Patients whose tumors had PD-L1 IHC 2+ (moderate to strong membrane staining in ≥ 25% of tumor cells) enjoyed longer LRFFS (5-year 100% vs. 74.4%, Hazard ratio [HR], 0.159, 95% confidence interval [CI], 0.021–0.988; *P* = 0.042) and marginally longer PFS (5-year 95.0% vs. 65.2%, HR, 0.351, 95% CI, 0.08–0.999, *P* = 0.067) compared to those whose tumors had PD-L1 IHC 0 (minimal membrane staining with PD-L1 in < 5% tumor cells or no staining with PD-L1) or 1+ (minimal to moderate membrane staining with PD-L1 in between 5–24% tumor cells). PD-L1 IHC 2+ was independently prognostic of both LRFFS (*P* = 0.014) and PFS (*P* = 0.045) in multivariable analyses. Only induction chemotherapy followed by concurrent chemoradiation was prognostic of DMFS (*P* = 0.003) and no prognostic factor for OS was identified.

**Conclusion:**

PD-L1 expression levels correlated with LRRFS and PFS in non-metastatic NPC treated with radical IMRT. It may play a role in radiosensitivity for NPC, which should be further confirmed in prospective studies using immunotherapy together with IMRT.

## Introduction

Nasopharyngeal carcinoma (NPC) is an endemic malignancy in southern China, Hong Kong, Taiwan and Singapore where there is a strong genetic predisposition [1,2]. The undifferentiated type (WHO Type 3) is highly associated with Epstein-Barr virus (EBV) which carries a better prognosis as compared to the keratinizinig squamous carcinoma (WHO Type 1). Radiation therapy is the mainstay of treatment for non-metastatic NPC especially for early-stage diseases while concurrent cisplatin-based chemoradiation is indicated for stage III to IVB diseases [3]. Intensity-modulated radiation therapy (IMRT) has been considered the standard radiation technique for NPC in virtue of its superior tumor coverage and organ sparing from necessary radiation leading to better locoregional control and reduced toxicities [4–10]. Unlike other head and neck squamous cell carcinoma in which anti-epidermal growth factor receptor (anti-EGFR) antibody cetuximab can be effectively combined with radiation therapy as curative treatment with similar efficacy as chemotherapy for non-metastatic diseases, there has been no conclusive evidence of targeted therapy combined with radiation therapy which showed comparable efficacy as chemotherapy in the same setting [11–14]. Recently, immune checkpoints and immunotherapy have been extensively studied in an attempt to redirect host anti-tumor responses to cancer cells [15]. It is well-recognized that cancer cells can evade host immunosurveillance by inhibiting functions of cytotoxic T-lymphocytes through the expression of certain ligands. Blockage of these ligands or their receptors by specific targeted drugs may result in reactivation of host immune responses thus enhancing anti-tumor effects. Programmed death-1 (PD-1) is an immune checkpoint on the surface of T-lymphocytes [16]. The corresponding ligand called programmed death-ligand 1 (PD-L1) is moderately to strongly expressed in various types of cancer including melanoma, non-small-cell lung cancer and head and neck cancers. Unlike EGFR in which high expression is associated with poor response to radiation therapy in head and neck cancers [17,18], PD-L1 expression has not been found to correlate with treatment outcomes after radiation therapy for head and neck cancers including NPC. Based on the above, we investigated the correlation of expression levels of PD-L1 in tumor cells and tumor infiltrating lymphocytes (TIL) with survival outcomes in patients with non-metastatic NPC treated with IMRT.

## Methods and Materials

### Patient eligibility and pretreatment investigations

Prior approval from local institutional review board (Institutional Review Board of the University of Hong Kong/Hospital Authority Hong Kong West Cluster, approval number UW 15–222) was obtained before study commencement. All clinical investigations were conducted according to the principles of Declaration of Helsinki. All patients provided written informed consent before study recruitment. Patients with previously untreated non-metastatic NPC who were treated with IMRT between 2005 and 2009 were reviewed from a prospectively collected database. Formalin-fixed paraffin-embedded (FFPE) archived nasopharyngeal tumor samples were retrieved for PD-L1 expression analysis as described below. Pretreatment investigations and workup included blood tests for serum hematology, biochemistry, hepatitis B serology, serology for antibodies against EBV viral capsid antigen (VCA) and early antigen (EA), fabrication of customized thermoplastic head and neck cast for the following contrast-enhanced computed tomography (CT) scanning of the head and neck region in IMRT treatment position with 3mm slice thickness co-registered with the T1, T2 and gadolinium-enhanced magnetic resonance images (MRI) of the head and neck region acquired by a 3-tesla scanner for subsequent target and organs-at-risk (OAR) delineation and subsequent IMRT treatment planning and optimization and contrast-enhanced CT scan of the thorax and abdomen to rule out distant metastases. Patients who suffered from stage I to small stage II diseases received IMRT alone while those with bulky stage II disease received concurrent chemoradiation with cisplatin 100mg/m^2^ on day 1, 22 and 43 of IMRT. Patients who suffered from stage III to IVB diseases received concurrent chemoradiation as mentioned followed by adjuvant chemotherapy with cisplatin 80mg/m^2^ on day 1 and 5-FU 1000mg/m^2^ from day 1 to 4 every 4 weeks for 3 more cycles starting 4 weeks after completion of IMRT, according to Intergroup 0099 trial [3, 19]. Some patients received 3 cycles of induction chemotherapy (cisplatin 100mg/m^2^ on day 1 and 5-FU 1000mg/m^2^ from day 1 to 5 every 3 weeks) before concurrent chemoradiation due to close proximity of their tumors to the normal critical OARs including brainstem, spinal cord, optic chiasm/nerves, etc, in an attempt for good tumor shrinkage so that a radical radiation dose could be delivered to the tumors with relative sparing of these OARs.

The gross tumor volumes of both the primary tumor (GTV-P) and the radiologically involved cervical nodes (GTV-N) were outlined on the planning CT images with the aid of co-registered MRI images as we described before [20]. Subsequently clinical target volume (CTV-70) and planning target volume containing CTV-70 with a 5mm margin (PTV-70) were generated to take into account the microscopic disease spread, physiological body motions and set-up errors respectively. Another CTV-66 encompassing the high risk areas including the posterior half of the maxillary sinuses, nasal cavities, parapharyngeal spaces, styloid processes, basiocciput, basisphenoid, clivus, foramina rotunda and ovale, pterygopalatine fossae, pterygomaxillary fissures, infra-orbital fissures, cavernous sinuses and level Ib and level V nodal stations was also contoured. A corresponding PTV-66 with a 3mm margin encompassing CTV-66 was created by boolean operations of the treatment planning system (Eclipse version 8.0, USA) which was also used for IMRT planning. OARs included brainstem, spinal cord, globes, optic nerves, optic chiasm, lenses, temporomandibular joints, temporal lobes, auditory nerves, cochleae, mandible, oral cavity, larynx, parotid glands and the vestibules [20]. During IMRT optimization, the maximum dose of brainstem, optic nerves and chiasm must be ≤ 54 Gy (allowing 0.1cc brainstem < 60Gy) and spinal cord ≤ 45Gy (allowing 0.1cc spinal cord < 48Gy). Efforts were also made to limit mean dose of parotid glands to 26 Gy whenever possible and dose to the lenses and temporal lobes as low as reasonably achieved without compromising dose coverage to the PTVs.

**Fig 1:**
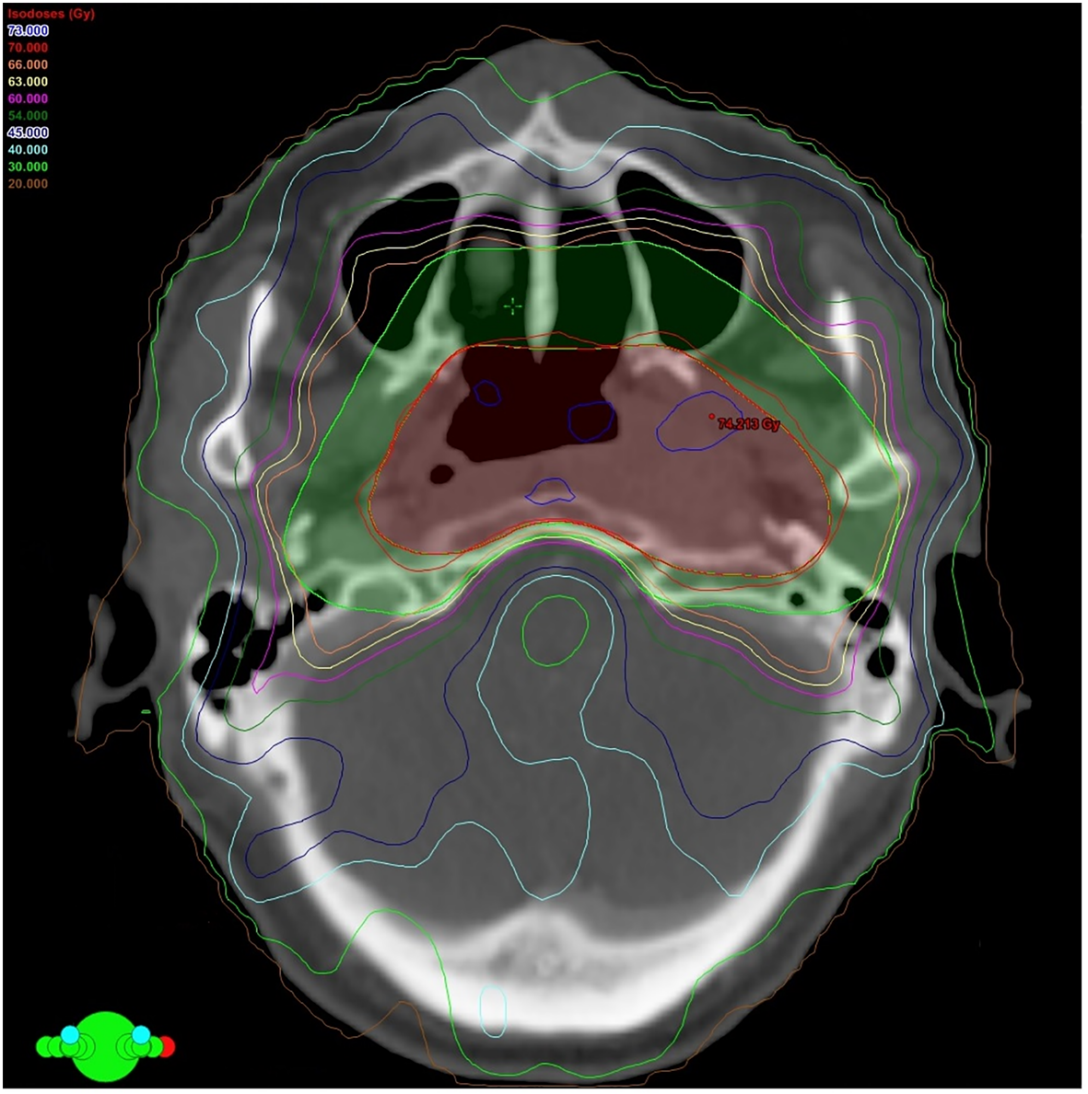
Computed tomography image showing the beam orientation and isodose distribution of a 9-field intensity-modulated radiation therapy (IMRT) plan for a patient with non-metastatic nasopharyngeal carcinoma.

A 9-field IMRT plan delivered by step-and-shoot technique was generated by Eclipse Treatment Planning System version 8.9, Palo Alto, CA, USA) using Anisotropic Analytical Algorithm (Fig 1). A total dose of 70 Gy and 66 Gy was prescribed to PTV-70 and PTV-66 respectively with simultaneous accelerated radiation therapy technique (SMART), as this has been the standard IMRT prescriptions in our institution for the past 10 years [20]. All IMRT plans fulfilled acceptance criteria with at least 95% of PTVs having received the prescribed dose, the maximum dose of PTVs limited to 107% or below and the maximum dose of organs-at-risk within tolerance limits according to International Commission on Radiation Units and Measurements (ICRU) criteria and they were delivered by a 6MV linear accelerator (Varian Medical Systems, Palo Alto, CA, USA). Positional verification with on-board imaging was performed before IMRT commencement. It was repeated again daily immediately before the first 3 fractions of IMRT followed and then weekly afterwards during the whole course of IMRT, to track any anteroposterior and lateral body displacements. All patients received regular clinical follow up and imaging surveillance every 3 to 4 months for the first year after IMRT, then every 6 months for the second year and yearly afterwards to exclude treatment failure and relapse.

### PD-L1 expression level by immunohisotchemistry

Immunohistochemical staining of 5μm sections from FFPE archived tissue of nasopharyngeal biopsies or excision specimens of tumor was performed by in the diagnostic laboratory in Anatomical Pathology Unit of our hospital with the antibody 1:200 anti-PD-L1 (E1L3N, Cell Signaling Technology®, Danvers, MA, USA) using the standard protocol for routine diagnostic specimens. Hematoxylin and eosin (H&E) sections were also reviewed for the presence of tumors and TIL. Scoring of the intensity and percentage of tumor cells were done semi-quantitatively. The intensity of immunohistochemical staining with PD-L1 was scored by a 3-point scale. Score 0 meant minimal membrane staining with PD-L1 in less than 5% tumor cells, or no staining with PD-L1 was identified. Score 1 (1+) indicated minimal to moderate membrane staining with PD-L1 in between 5% to 24% tumor cells while score 2 (2+) comprises more positivity defined as at least 25% tumor cells demonstrating moderate to strong complete membrane staining with PD-L1. The same scoring system also applied to PD-L1 expression for TILs but their IHCs were reported separately from the IHCs for tumor cells.

### Statistical analysis

Kaplan-Meier methods were employed for survival studies. Log-rank tests were used comparison of survival outcomes among patients with different stages of NPC and different expression levels of PD-L1 as defined above. Cox proportional hazard models with univariable and multivariable analyses were performed to identify prognostic factors of survival endpoints including locoregional failure-free survival (LRFFS), progression-free survival (PFS), distant metastasis-free survival (DMFS) and overall survival (OS) with age as continuous covariate and sex, use of IMRT alone, use of concurrent chemoradiation alone, use of induction chemotherapy followed by concurrent chemoradiation, use of concurrent chemoradiation followed by adjuvant chemotherapy, T-classification (T1/T2 vs. T3/T4), N-classification (N0/N1 vs. N2/N3), overall stage (I/II vs. III/IVA-B), expression levels of PD-L1 as categorical covariates. LRFFS was calculated from the starting date of IMRT to the date of occurrence of locoregional recurrence or death from any cause. PFS started from date of commencement of IMRT to the date of disease progression or death from any cause. DMFS was calculated from the starting date of IMRT to the date of occurrence of distant metastasis or death from any cause. OS was defined from the date of commencement of IMRT to the date of death from any cause. Receiver-operating characteristic (ROC) curves were constructed for the performance of the cut-off value of IHC scores of PD-L1 expression on the survival endpoints. Statistical significance was defined as *P* < 0.05 (two-sided). All statistical analyses were performed by Statistical Package for Social Sciences (SPSS) version 20.0.

## Results

Out of 146 consecutive patients with non-metastatic NPC treated with radical IMRT, only 104 patients were eligible with adequate archived tumor samples for IHC staining and subsequent analysis. The baseline characteristics of these 104 patients were displayed in Table 1. Fourteen patients (13.5%) received IMRT alone because of early-stage (stage I and II) diseases, advanced age or significant medical comorbidities. Seventeen patients (16.3%) received concurrent chemoradiation alone for their bulky stage II disease. Twenty patients (19.2%) received induction chemotherapy followed by concurrent chemoradiation at the discretion of treating radiation oncologists because of the close proximity of their primary tumors to the critical OARs. The remaining 53 patients (51.0%) received concurrent chemoradiation followed by adjuvant chemotherapy. All patients completed IMRT without suspension.

**Table 1:** Baseline patient characteristics of the 104 patients.

Characteristic	N (%)
Median age in years (range)	53 (27–80)
Male/female	85 (81.7)/19 (18.3)
T-classification	
T1	24 (23.1)
T2	30 (28.8)
T3	39 (37.5)
T4	11 (10.6)
N-classification	
N0	26 (25.0)
N1	25 (24.0)
N2	41 (39.4)
N3a	6 (5.8)
N3b	6 (5.8)
Overall stage	
I	12 (11.5)
II	20 (19.2)
III	50 (48.1)
IVA	10 (9.6)
IVB	12 (11.5)
IMRT alone	14 (13.5)
Concurrent chemoradiation	
Concurrent chemoradiation alone	17 (16.3)
Induction chemotherapy then concurrent chemoradiation	20 (19.2)
Concurrent chemotherapy then adjuvant chemotherapy	53 (51.0)
PD-L1 expression level by immunohistochemistry	
0	78 (75.0)
1+	4 (3.8)
2+	22 (21.2)

Abbreviations: IMRT, intensity-modulated radiation therapy; PD-L1, programmed death-ligand 1.

After a median follow-up duration of 7.6 years (5.7–10.8 years), 21 (20.2%), 19 (18.3%) and 31 (29.8%) patients suffered from locoregional failure, distant metastases and overall disease progression respectively and 31 (29.8%) patients died during the study period. The median LRFFS, PFS, DMFS and OS were not reached. The mean LRFFS, PFS, DMFS and OS were 78.5, 71.7, 94.0 and 89.4 months respectively. The 5-year corresponding rates were 79.9%, 71.4%, 85.7% and 81.7% respectively.

### Patterns of IHC staining with PD-L1

**Fig 2:**
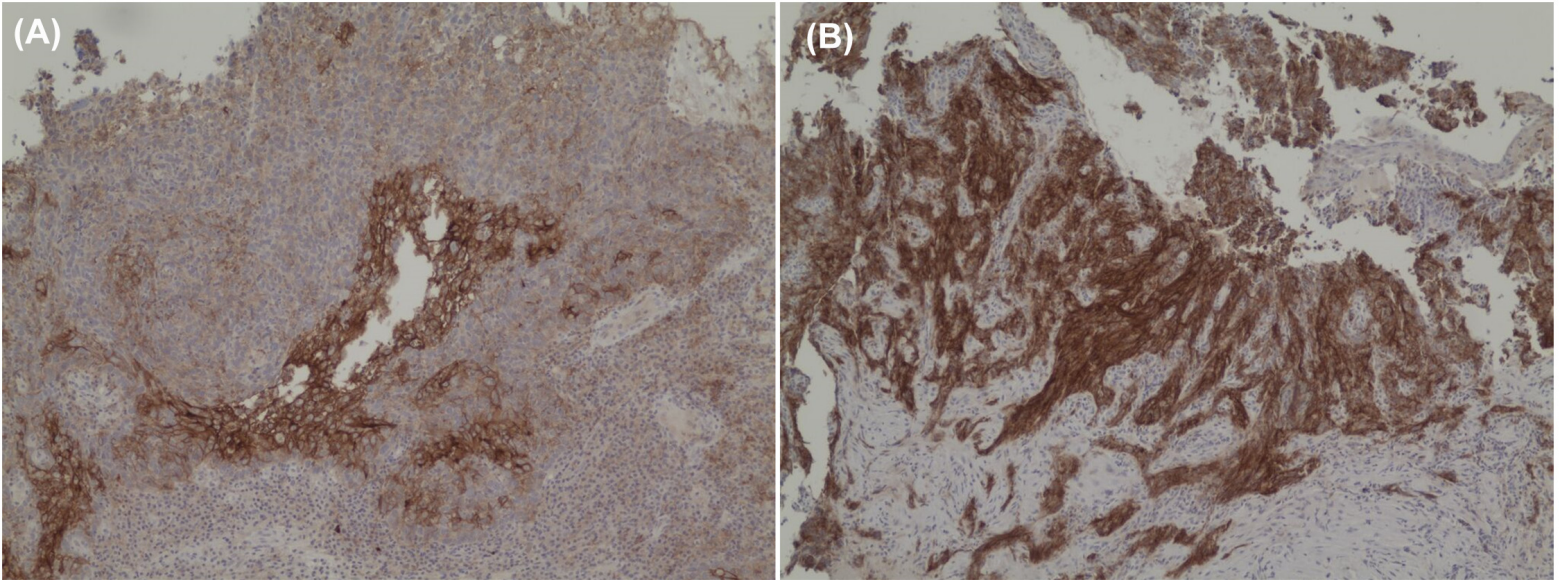
Immunohistochemical staining for PD-L1 in patients with non-metastastic nasopharyngeal carcinoma. (A) A patient whose tumor was scored 1+ (weak to moderate membrane in about 5% of tumor cells) on immunohistochemical staining with PD-L1. (B) Another patient whose tumor was scored 2+ (moderate to strong membrane staining in more than 25% tumor cells) on immunohistochemical staining with PD-L1.

**Fig 3:**
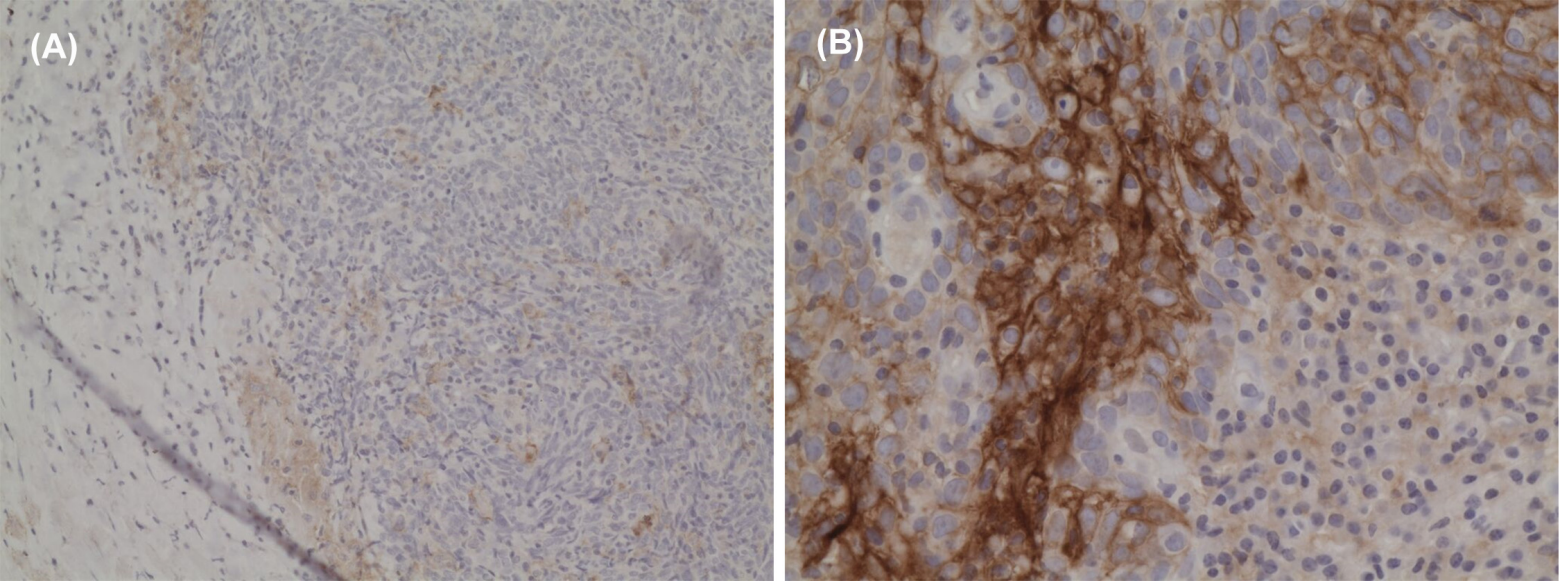
Micrograph showing the absence of immunohistochemical staining for PD-L1 in tumor-infiltrating lymphocytes. (A) Low-power field. (B) High-power field despite strong immune-positivity to PD-L1 in the adjacent tumor cells.

Seventy-eight patients (75.0%) had no membrane staining with PD-L1 for their tumor cells. Four (3.8%) and 22 (21.2%) patients had IHC 1+ and IHC 2+ for their tumor cells respectively (Fig 2). The baseline clinical demographics between these 2 groups of patients were shown, without any statistically significant differences noted (Table 2). This observation suggested that PD-L1 expression might not be common in NPC tumor cells. However, once the gene was induced, expression level can be prominent and in significant proportion of the tumor. Therefore we grouped IHC 0 or 1+ as one group against IHC 2+ in our subsequent univariable and multivariable analyses as we believed that tumour cells with IHC 1+ (minimal to moderate membrane staining) behaved similarly as those with IHC 0. Interestingly, although there were many TILs associated with the tumor cells, PD-L1 was only expressed in these tumor-associated lymphoid cells in a scattered manner. None of the tumors had more than 10% of intimately associated lymphocytes with discernible PD-L1 staining and thus all TILs scored 0 for their PD-L1 expression (Fig 3).

**Table 2:** Comparison of baseline clinical parameters of patients stratified by the levels of PD-L1 expression with immunohistochemical staining (0 or 1+ versus 2+).

	IHC 0 or 1+	IHC 2+	*P*
Median age in years (range)	52.5 (27–80)	53.5 (30–75)	0.855
Male/female	66/16	19/3	0.527
T-classification			0.523
T1	19	5	
T2	21	9	
T3	33	6	
T4	9	2	
N-classification			0.117
N0	23	3	
N1	22	3	
N2	29	12	
N3a	3	3	
N3b	5	1	
Overall stage			0.865
I	10	2	
II	16	4	
III	40	10	
IVA	8	2	
IVB	8	4	
IMRT alone	12	2	0.499
Concurrent chemoradiation			0.793
Concurrent chemoradiation alone	13	4	
Induction chemotherapy then concurrent chemoradiation	16	4	
Concurrent chemotherapy then adjuvant chemotherapy	41	12	

Abbreviations: IHC, immunohistochemical staining; IMRT, intensity-modulated radiation therapy.

**Fig 4:**
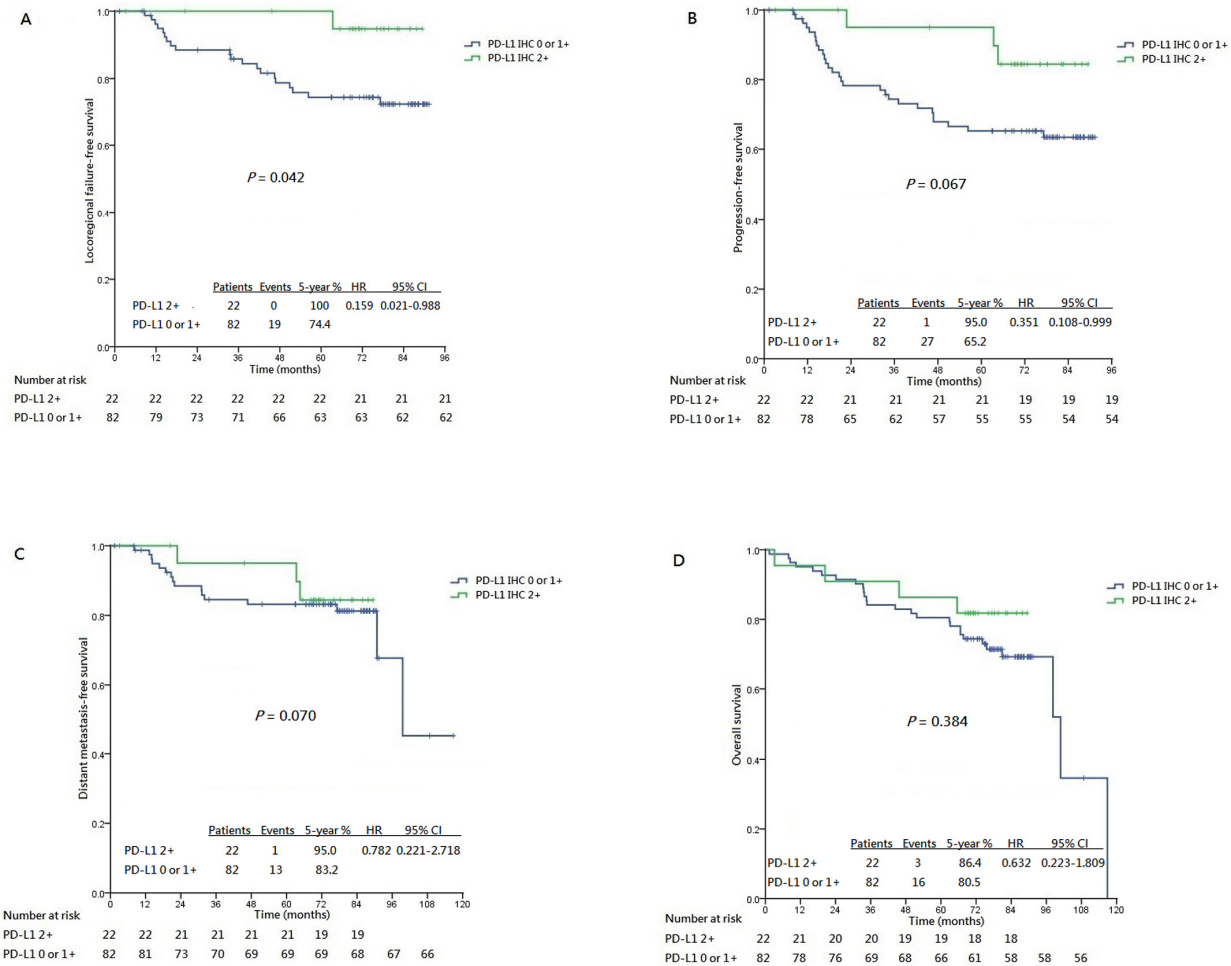
Kaplan-Meier curves stratified by the patterns of immunohistochemical staining for PD-L1 (2+ vs 0 or 1+). (A) Loco-regional failure-free survival. (B) Progression-free survival. (C) Distant metastasis failure-free survival. (D) Overall survival.

**Fig 5:**
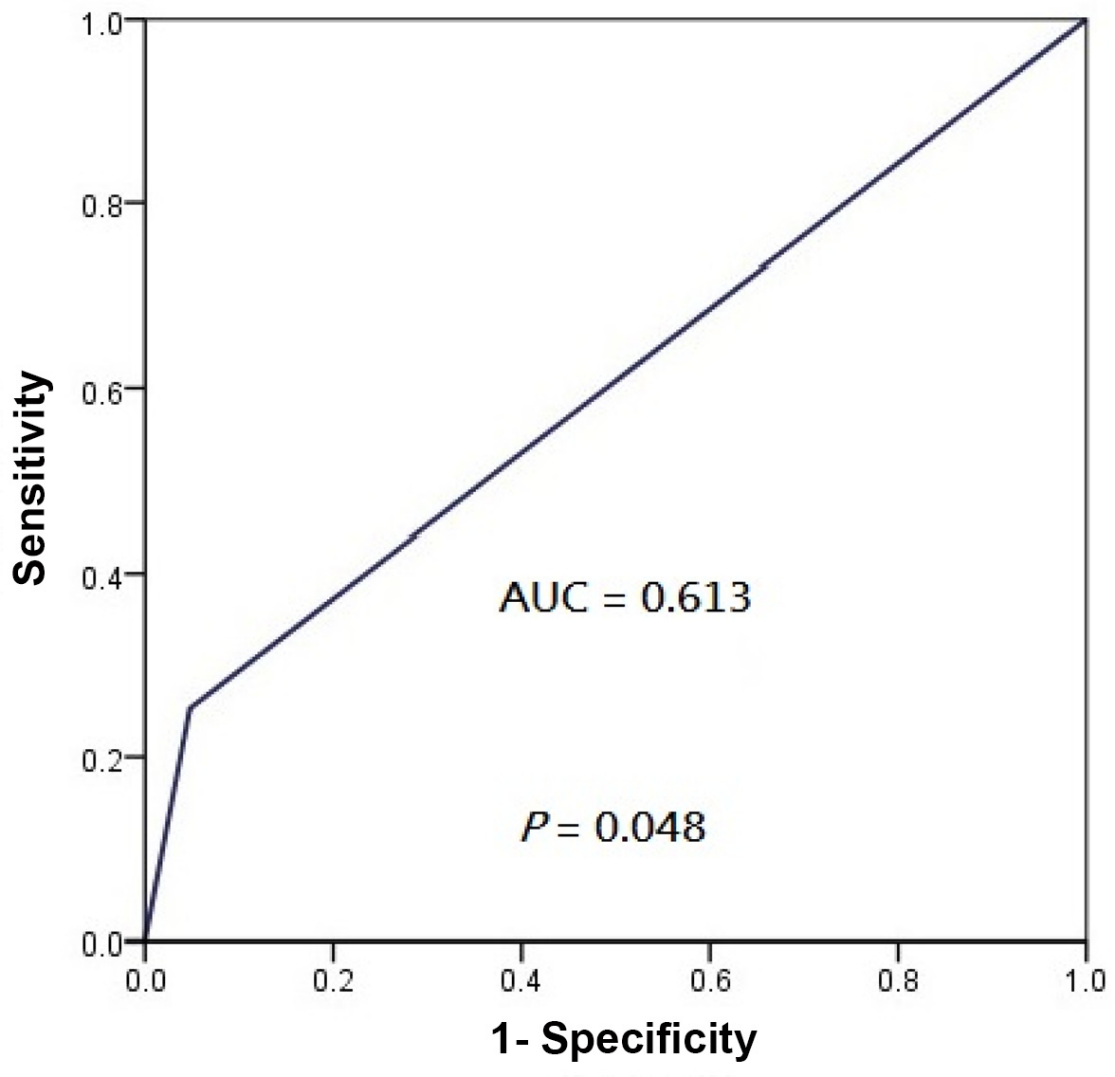
Receiver operating characteristics curve showing the performance of PD-L1 IHC 2+ as cut-off for locoregional progression of patients with non-metastatic NPC treated with radical intensity-modulated radiation with or without adjunct chemotherapy.

**Fig 6:**
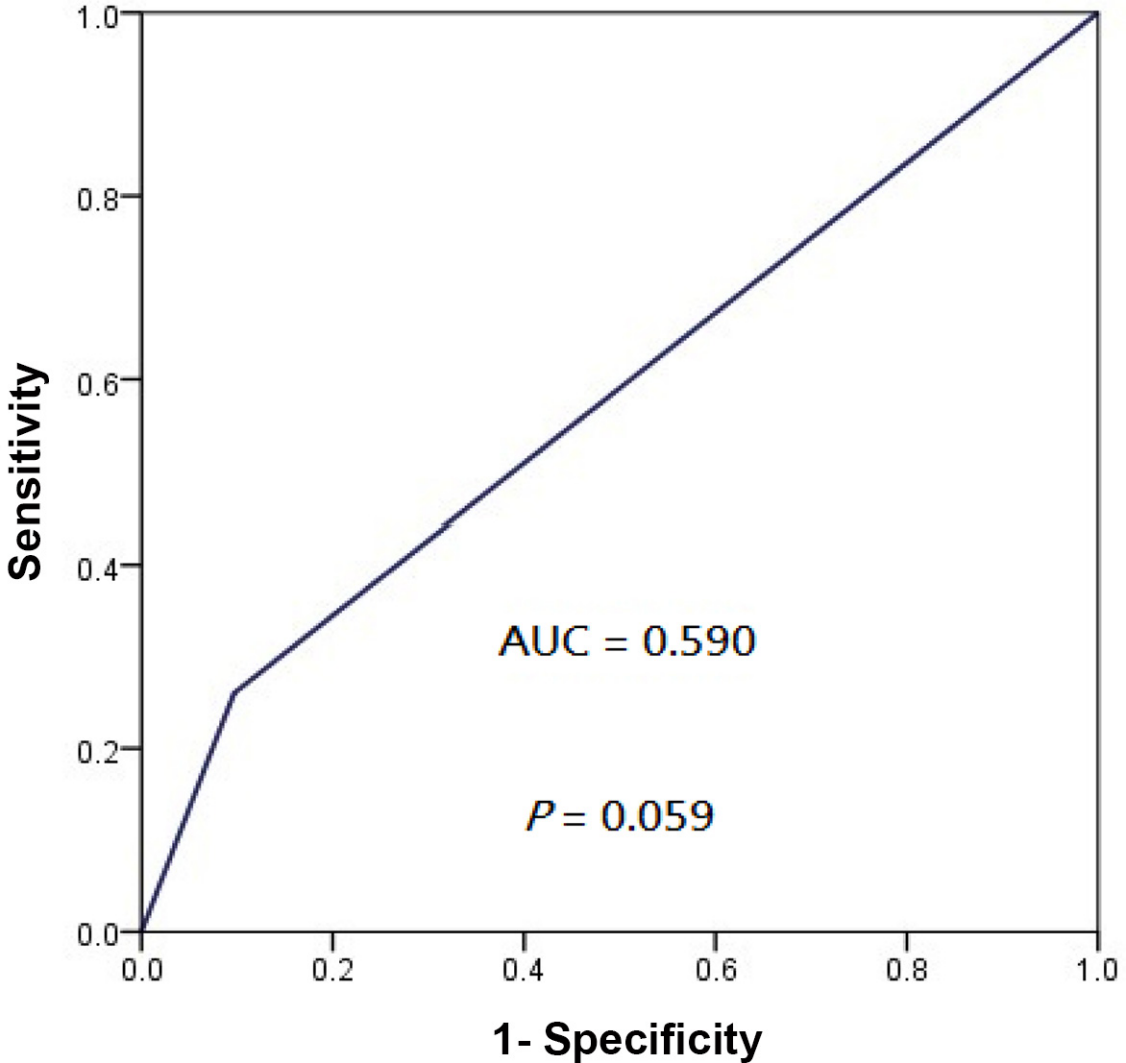
Receiver operating characteristics curve showing the performance of PD-L1 IHC 2+ as cut-off for overall progression of patients with non-metastatic NPC treated with radical intensity-modulated radiation with or without adjunct chemotherapy.

Subgroup analyses revealed that patients whose tumors exhibited PD-L1 IHC 2+ enjoyed a significantly longer LRFFS (5-year 100%, hazard ratio [HR], 0.159, 95% confidence interval [CI], 0.021–0.988; *P* = 0.042) compared to those whose tumors were PD-L1 IHC 0 or 1+ only (5-year 74.4%) (Fig 4A). Similarly, marginally longer PFS was also seen in those whose tumors exhibited PD-L1 IHC 2+ (5-year 95.0%, HR, 0.351, 95% CI, 0.108–0.999; *P* = 0.067) compared to those whose tumors was PD-L1 0 or 1+ only (5-year 65.2%) (Fig 4B). No significant differences in DMFS and OS were noticed between patients whose tumors exhibited PD-L1 IHC 2+ and the rest whose tumors exhibited PD-L1 IHC 0 or 1+ only (Fig 4C and 4D), despite a numerical advantage for patients whose tumors demonstrating PD-L1 IHC 2+. ROC analysis showed that the area-under-the-curve (AUC) values for locoregional progression and overall progression was 0.613 (95% CI, 0.479–0.726, *P* = 0.048) and 0.590 (95% CI, 0.467–0.697, *P* = 0.059) respectively by using IHC 2+ as the cutoff (Figs 5 and 6).

### Univariable and multivariable analyses

Univariable and multivariable analyses were performed for LRFFS and PFS (Tables 3 and 4) as well as DMFS and OS (Tables 5 and 6). IMRT alone versus concurrent chemoradiation was not prognostic of any survival endpoint. While T-classification, induction chemotherapy followed by concurrent chemoradiation, PD-L1 IHC 0 or 1+ and PD-L1 IHC 2+ were significant in univariable analysis, only induction chemotherapy followed by concurrent chemoradiation (*P* = 0.029) and PD-L1 IHC 2+ (*P* = 0.014) were independently and significantly prognostic of LRFFS in multivariable analysis. For PFS, T-classification, induction chemotherapy followed by concurrent chemoradiation and PD-L1 IHC 0 or 1+ and PD-L1 IHC 2+ were significant in univariable analysis. However only T-classification (*P* = 0.019) and PD-L1 IHC 2+ (*P* = 0.045) were significant as independent prognostic factors in the subsequent multivariable analysis. For DMFS, only induction chemotherapy followed by concurrent chemoradiation was prognostic in both univariable (*P* = 0.003) and multivariable analyses (*P* = 0.003). Only T-classification was prognostic of OS in both univariable (*P* = 0.031) and multivariable analyses (*P* = 0.026). Besides, no statistical analysis was performed for the correlation between PD-L1 expression of TIL and each survival endpoint because no definite PD-L1 staining was observed for all TILs.

**Table 3:** Univariable and multivariable analysis for locoregional failure-free survival.

	Univariable analysis			Multivariable analysis^a^		
	HR	95% CI	*P*	HR	95% CI	*P*
Age	0.976	0.940–1.014	0.216	ND		
Sex (male as reference)	1.328	0.391–4.512	0.650	ND		
T-classification						
T1/T2 vs. T3/T4	0.388	0.156–0.964	0.042	0.465	0.268–1.254	0.162
N-classification						
N0N1 vs. N2N3	0.737	0.310–1.749	0.488	ND		
Overall stage						
I/II vs. III/IVA-B	0.307	0.090–1.043	0.058	0.415	0.125–1.297	0.161
IMRT alone	3.546	0.476–26.316	0.217	ND		
Concurrent chemoradiation	0.456	0.106–1.961	0.291	ND		
Induction chemotherapy then concurrent chemoradiation	0.324	0.134–0.785	0.023	0.337	0.136–0.802	0.029
Concurrent chemoradiation then adjuvant chemotherapy	1.069	0.454–2.518	0.879	ND		
PD-L1 expression level						
PD-L1 1+	0.268	0.062–1.152	0.077	0.278	0.061–1.149	0.060
PD-L1 2+	0.162	0.022–1.000	0.042	0.159	0.021–0.988	0.014

Abbreviations: CI, confidence interval; HR, hazard ratio; IMRT, intensity-modulated radiation therapy; ND, not done; PD-L1, programmed death-ligand 1.

^a^Only covariates found significant (*P* < 0.1) in the univariable analysis were considered in the multivariable analysis.

**Table 4:** Univariable and multivariable analyses for progression-free survival.

	Univariable analysis			Multivariable analysis^a^		
	HR	95% CI	*P*	HR	95% CI	*P*
Age	0.992	0.963–1.022	0.606	ND		
Sex (male as reference)	0.899	0.368–2.194	0.815	ND		
T-classification						
T1/T2 vs. T3/T4	0.357	0.168–0.759	0.007	0.363	0.170–0.761	0.009
N-classification						
N0/N1 vs. N2/N3	0.708	0.347–1.446	0.343	ND		
Overall stage						
I/II vs. III/IVA-B	0.437	0.179–1.066	0.069	0.456	0.176–1.131	0.110
IMRT alone	2.632	0.628–10.989	0.186	ND		
Concurrent chemoradiation	0.643	0.225–1.838	0.410	ND		
Induction chemotherapy then concurrent chemoradiation	0.335	0.160–0.702	0.014	0.401	0.205–1.563	0.254
Concurrent chemoradiation then adjuvant chemotherapy	0.820	0.404–1.664	0.582	ND		
PD-L1 expression level						
PD-L1 1+	0.386	0.135–1.104	0.076	0.398	0.140–1.108	0.056
PD-L1 2+	0.346	0.105–1.010	0.067	0.351	0.108–0.999	0.045

Abbreviations: CI, confidence interval; HR, hazard ratio; IMRT, intensity-modulated radiation therapy; ND, not done; PD-L1, programmed death-ligand 1.

^a^Only covariates found significant (*P* < 0.1) in the univariable analysis were considered in the multivariable analysis.

**Table 5:** Univariable and multivariable analysis for distant metastasis-free survival.

	Univariable analysis			Multivariable analysis^a^		
	HR	95% CI	*P*	HR	95% CI	*P*
Age	1.006	0.968–1.045	0.775	ND		
Sex (male as reference)	0.676	0.241–1.896	0.457	ND		
T-classification						
T1/T2 vs. T3/T4	0.392	0.148–1.034	0.058	0.495	0.183–1.335	0.165
N-classification						
N0N1 vs. N2/N3	0.486	0.188–1.255	0.136	ND		
Overall stage						
I/II vs. III/IVA-B	0.550	0.181–1.670	0.291	ND		
IMRT alone	2.899	0.386–21.739	0.301	ND		
Concurrent chemoradiation	1.060	0.304–3.689	0.927	ND		
Induction chemotherapy then concurrent chemoradiation	0.217	0.083–0.568	0.003	0.219	0.080–0.565	0.003
Concurrent chemoradiation then adjuvant chemotherapy	0.472	0.177–1.263	0.135	ND		
PD-L1 expression level						
PD-L1 1+	0.533	0.155–1.835	0.319	ND		
PD-L1 2+	0.782	0.225–2.725	0.700	ND		

Abbreviations: CI, confidence interval; HR, hazard ratio; IMRT, intensity-modulated radiation therapy; ND, not done; PD-L1, programmed death-ligand 1.

^a^Only covariates found significant (*P* < 0.1) in the univariable analysis were considered in the multivariable analysis.

**Table 6:** Univariable and multivariable analyses for overall survival.

	Univariable analysis			Multivariable analysis		
	HR	95% CI	*P*	HR	95% CI	*P*
Age	1.068	0.968–1.100	0.975	ND		
Sex (male as reference)	0.985	0.398–2.439	0.974	ND		
T-classification						
T1/T2 vs. T3/T4	0.432	0.201–0.928	0.031	0.429	0.211–0.919	0.026
N-classification						
N0/N1 vs. N2/N3	0.810	0.393–1.667	0.567	ND		
Overall stage						
I/II vs. III/IVA-B	0.428	0.162–1.125	0.085	0.452	0.189–1.273	0.145
IMRT alone	2.101	0.499–8.850	0.311	ND		
Concurrent chemoradiation	0.534	0.161–1.767	0.304	ND		
Induction chemotherapy then concurrent chemoradiation	0.965	0.365–2.551	0.943	ND		
Concurrent chemoradiation then adjuvant chemotherapy	0.532	0.245–1.152	0.109	ND		
PD-L1 expression level						
PD-L1 1+	0.576	0.220–1.508	0.261	ND		
PD-L1 2+	0.627	0.217–1.812	0.388	ND		

Abbreviations: CI, confidence interval; HR, hazard ratio; IMRT, intensity-modulated radiation therapy; ND, not done; PD-L1, programmed death-ligand 1.

^a^Only covariates found significant (*P* < 0.1) in the univariable analysis were considered in the multivariable analysis.

## Discussion

In our study, PD-L1 was overexpressed in 25% (26 of 104 patients) of NPC patients and 21.2% of our patients whose tumors were IHC 2+ with PD-L1. Our result revealed that PD-L1 IHC 2+ was associated with better LRRFS and PFS. These advantages of PD-L1 expressed tumors have been observed in stage I resected pulmonary adenocarcinoma which was associated with a better relapse-free survival [21]. In a previous study by Zhang et al, PD-1 alone, an immune checkpoint expressed on the surface of T-lymphocytes upon activation, or in combination with PD-L1, was associated with a worse disease-free survival in NPC patients [22]. However this study also included stage IVC metastatic diseases and their patients were all treated with conventional radiation technique. As the latter radiation modality was considered outdated in the modern era of IMRT or other precision radiation technique, their conclusion on the prognostic significance of PD-L1 expression was not directly comparable to our current observations. Our study, on the contrary, recruited a more representative population of non-metastatic NPC patients who were all treated with standard dosing schedules of IMRT. On the other hand, other clinical parameters for instance IMRT alone versus concurrent chemoradiation and the sequence of chemotherapy (except induction chemotherapy followed by concurrent chemoradiation which was prognostic of LRRFS and DMFS and advanced T-classification which was prognostic of PFS and OS) were not prognostic factors of the survival endpoints.

The role of TILs on survival outcome should be further explored. Hsu et al demonstrated that the presence of PD-1 expressing TILs was prognostic of locoregional relapse-free survival, disease-free survival and OS [23]. However only 46 patients were analyzed in their study, which were fewer than our study population. In addition, the definition of PD-1 positivity in both the tumor cells and TILs was not clearly stated. Besides, the chosen cut-off value of median PD-1 expression rate set at 27.8% was rather arbitrary and haphazard without detailed explanation. Instead, all TILs virtually did not express PD-L1 in our study and their prognostic role needs further investigation in future studies.

The prognostic role of PD-L1 in various types of malignancies remains to be deciphered. Though most studies delineated negative correlation of PD-L1 expression with survival in general [24–28], other studies in specific tumors such as colorectal cancer, non-small cell lung cancer and melanoma demonstrated more favorable correlations between its expression and survival [29–32]. This reflected that the interaction between PD-L1 expression and host immune responses differs among various types of cancer. In fact, PD-L1 expression is not directly proportional to tumor immune evasion and it may just signify an ongoing anti-tumor immune response that leads to production of interferon-γ and other inflammatory markers [32]. The tumor microenvironment may be even more complication in virus-associated tumors like NPC, as neoantigens associated with infection by these tumor-promoting viruses may give rise to inflammatory responses leading to PD-L1 expression [29,33]. In addition, whether it has another role on radiosensitivity or radioresistance for head and neck cancer and NPC warrants further studies for confirmation.

The scoring system of IHC for PD-L1 has yet to be defined and it may vary for different types of malignancies. Traditionally, the median value of the percentage of tumor cells positive for PD-L1 in order to balance the patient groups is most often used. Alternatively, the semiquantitative H-score, obtained by multiplying the percentage of positively-stained tumor cells and the intensity of staining was also commonly used. In addition, definition of staining positivity (for example cell surface versus cytoplasmic expression, by tumor cells only or by other cells in the tumor milieu, percentage of stained cells regarded as positive) has not reached a unanimous consensus. For instance, positivity defined as ≥ 5% tumor cells positively stained with PD-L1 has recently gained popularity in lung cancer studies [21,34]. In fact even for phase III randomized-controlled trials using the FDA-approved pembrolizumab and nivolumab for advanced non-small cell lung cancer, the cutoffs (1% and 50% for pembrolizumab in their KEYNOTE-001) and (1%, 5% and 10% for nivolumab in their CheckMate 017 trial) chosen were arbitrary at the drug sponsor’s discretion without detailed explanation and their subsequently statistical analyses were also exploratory without detailed elaboration [35–37]. The percentage of PD-L1 positivity was also heavily dependent on the performance of the test kits and antibodies, the number of slides reviewed and the amount of tumor cells and TIL retrieved during micro-dissection of the specimens which can be highly operator dependent. As a result, we thought that choosing 5% and 25% (instead of 1% and 5% in the drug-sponsored trials) as cutoffs were less susceptible to the effect of sampling size and sampling errors of the tumor cells retrieved during micro-dissection. The general impression of PD-L1 expression studies in various types of tumors suggested that different tumors would have different scoring systems. This might include not just differences in the percentage and intensity of PD-L1 expression in tumor cells to be considered positive or negative IHC results, but might extend to include PD-L1 expression in the TIL or even the stromal cells. In the context of immune checkpoints and immunotherapy, these observations suggested that consideration of the overall tumor and microenvironment should be important in predicting the success of immunotherapy. Ultimately, it would be most desirable if the definition and scoring system for PD-L1 IHC correlate with treatment response and survival.

## Conclusion

We conclude that PD-L1 expression level can be used as a prognostic factor of LRRFS and PFS in non-metastatic NPC patients treated with radical IMRT. This has provided insight in designing immunotherapy targeting the PD-L1 pathway in combination with IMRT in the future, which should be validated in prospective randomized studies.
